
JOURNAL INFORMATION
==============================
NLM Title Abbreviation: ScientificWorldJournal
Journal ID: ScientificWorldJournal
NLM Journal ID: 101131163
Journal ID: TSWJ

The Scientific World Journal

ISSN: 2356-6140
EISSN: 1537-744X
Publisher: Wiley

ARTICLE INFORMATION
==============================
PMCID: PMC6084093
PMID: 30147521
DOI: 10.1100/tsw.2001.172
Article ID: 821209
Article version: 1
Subjects: Short Report

Purification and Characterization of Apoptotic Factors from Human Milk

Semenov Dmitry V.
Kuligina Elena V.
Shestova Olga E.
Potapenko Miroslava O.
Romannikova Irina V.
Richter Vladimir A. *
Laboratory of Radiochemistry, Novosibirsk Institute of Bioorganic Chemistry, Siberian Branch of Russian Academy of Sciences, Lavrentiev Ave., 8, Novosibirsk, 630090, Russia
*Vladimir A. Richter: richter@niboch.nsc.ru
Publication date: 2001
Electronic publication date: 2001 Oct 17
Volume: 1
First page: 56
Last page: 56
Copyright: Copyright © 2001 Dmitry V. Semenov et al.
Copyright year: 2001
License: This is an open access article distributed under the Creative Commons Attribution License, which permits unrestricted use, distribution, and reproduction in any medium, provided the original work is properly cited.
License URL: https://creativecommons.org/licenses/by/3.0/

Funding: Russian Academy of Sciences

==============================
Miami *Nature Biotechnology* Short Reports
*TheScientificWorld* (2001) 1(S3), 56SR
ISSN 1532-2246; DOI 10.1100/tsw.2001.172

### PURIFICATION AND CHARACTERIZATION OF APOPTOTIC FACTORS FROM HUMAN MILK

Dmitry V. Semenov, Elena V. Kuligina, Olga E. Shestova, Miroslava O. Potapenko, Irina V. Romannikova, and Vladimir A. Richter*

Laboratory of Radiochemistry, Novosibirsk Institute of Bioorganic Chemistry, Siberian Branch of Russian Academy of Sciences, Lavrentiev Ave., 8, Novosibirsk, 630090, Russia.
* Richter@niboch.nsc.ru

**INTRODUCTION.** Human milk contains a wide array of biologically active compounds that influence tissue and organs development of children and control process of lactation of mothers. Resent studies have provided evidence that human milk induced programmed cell death (PCD) in a variety of transformed and immature mammalian cells. The active component was identified as multimeric form of α-lactalbumin (1). Conversion of native α-lactalbumin to a folding variant with cytotoxic capacity was achieved with special conditions of isolation (2). α-Lactalbumin is the most abundant protein of human milk and it is presented in milk in monomeric form (1, 2). Therefore, nothing is known about apoptotic components of whole human milk. In order to determine what milk components are capable to induce apoptosis we worked out the method of their isolation and purification.

**METHOD.** Milk fractionation was performed using acid ethanol extraction and multistep chromatography purification. Influence of milk fractions on cell viability (MCF-7, SPEV, CV-1) was evaluated using the MTT assay according to standard procedure. Cell morphology was examined using fluorescence and visual spectra microscopy with propidiumiodide and Giemsa staining technique. Oligonucleosomal fragmentation of DNA was analysed by agarose gel electrophoresis.

**RESULTS AND DISCUSSION.** Human milk induced programmed cell death in all examined cell cultures. Our preliminary milk fractionation experiments have provided evidence that milk contained two different proteins capable to cause apoptosis. α-Lactalbumin contained fractions had no effect on cell morphology and cell viability.

Dominant PCD factor was strongly associated with β–casein fractions. But it could be completely separated from casein micelles with acid ethanol extraction (method developed for purification of insulin and TGF-β (3)). Minor PCD factor of human milk was found to be a low molecular weight oligopeptide. Dominant and minor factors caused different preapoptotic morphological changes in cell culture. The first one provided cell cycle arrest and appearance of vacuoles in cytosol of fibroblast cells (CV-1, SPEV). The second factor induced classical apoptotic changes of cell morphology.

The physico-chemical characteristics and the whole complex of morphological changes induced by the dominant milk factor into cells pointed out the likeness of this protein with transforming growth factor – β (TGF-β). In accordance with these data we worked out the isolation and purification method for milk apoptotic factors. Our method includes the acid ethanol extraction, ionexchange and reversed phase HPLC chromatography. However the final identification and purification of these factors remains to be carried out. Recent studies discover significant role of human milk TGF-β isoforms in epithelium apoptosis on the first stage of mammary gland involution and the importance of milk TGF-β as nutritional immunoregulator (4,5). These data and our observations emphasise the major role of such compounds in tissue development by controlling the cell survival.

**ACKNOWLEDGEMENT.** This work was supported by grant for young scientists from SB RAS Siberian Branch of the Russian Academy of Sciences.

**REFERENCES.**

1. Hakansson, A., Zhivotovsky, B., Orrenius, S., Sabharwal, H., and Svanborg, C. (1995) Proc. Natl. Acad. Sci. U S A 92, 8064-8068
2. Svensson, M., Hakansson, A., Mossberg, A.-K., Linse, S., and Svanborg, C. (2000) Proc. Natl. Acad. Sci. U S A 97, 8, 4221-4226
3. Roberts, A.B., Lamb, L.C., Newton, D.L., Spporn, M.B., DeLarco, J.E., and Torado, J.L. (1980) Proc. Natl. Acad. Sci. U S A 77, 3493-3498
4. Donnet-Hughes, A., Duc, N., Serrant, P., Vidal, K., and Schiffin, E.J. (2000) Immunol. Cell Biol. 78, 74-79
5. Nguyen, A.V. and Pollard, J.W. (2000) Development 127, 3107-3118
